# Structural Equation Modeling of Parkinson's Caregiver Social Support, Resilience, and Mental Health: A Strength-Based Perspective

**DOI:** 10.1155/2020/7906547

**Published:** 2020-02-14

**Authors:** Carmen M. Tyler, Richard S. Henry, Paul B. Perrin, Jack Watson, Teresita Villaseñor, Sarah K. Lageman, Erin R. Smith, Genoveva Rizo Curiel, Judith Avila, Miriam E. Jimenez Maldonado, Jose A. Soto-Escageda

**Affiliations:** ^1^Department of Psychology, Virginia Commonwealth University, Richmond, VA, USA; ^2^Department of Physical Medicine and Rehabilitation, Virginia Commonwealth University, Richmond, VA, USA; ^3^Hospital Civil Fray Antonio Alcalde, Guadalajara, Mexico; ^4^Department of Neurosciences, University of Guadalajara, Guadalajara, Mexico; ^5^Department of Neurology, Parkinson's & Movement Disorders Center, Virginia Commonwealth University, Richmond, VA, USA

## Abstract

Only scant literature has focused on social support in Parkinson's disease (PD) caregivers, and no studies to date have examined resilience in this population, despite both variables having been shown to be important in other caregiving populations. As a result, the purpose of the current study was to construct and validate a theoretical structural equation model whereby social support is associated with higher levels of resilience in PD caregivers and increased resilience is related to decreased mental health symptoms. Two hundred fifty three PD caregivers from two clinics in the United States and Mexico completed self-report measures of these constructs. Results suggested that the hypothesized pattern was robustly supported with the structural equation model showing generally good fit indices. Higher levels of social support were associated with increased resilience, which in turn was associated with reduced mental health symptoms. Resilience partially mediated social support's effect on mitigating mental health symptoms. The model explained 11% of the variance in resilience and 35% in mental health symptoms. These findings have implications for future research on the development and tailoring of interventions to improve social support, resilience, and mental health in PD caregivers.

## 1. Introduction

Parkinson's disease (PD) is a neurodegenerative disorder that gradually erodes a person's motor and cognitive functions, resulting in increasing disability; PD trails only Alzheimer's disease in neurodegenerative disorder occurrence, and risk for developing PD increases with age [1]. As people are living longer, the proportion of older people in global populations is increasing and rates of those who will develop PD are expected to rise accordingly [2], with incidence rates similar across the Americas [3]. Continued progression of PD requires increasing levels of assistance from others for the individual with Parkinson's disease (IWPD) [4], with caregiving responsibilities usually being undertaken by informal caregivers, often aging spouses or other family members [5, 6].

Caregiving for a person with a neurodegenerative disease has been shown to be a high-demand role which can adversely affect the physical and mental health and wellbeing of the caregiver (e.g., [7–10]). For example, providing higher levels of care has been linked to adverse caregiver outcomes, with caregivers who provide substantial levels of care (e.g., assisting with activities of daily living) having decreased mental health [11], significantly more chronic illnesses, and increased use of tranquilizers [8]. As the necessity for caregivers to assist with activities of daily living increases with PD symptom progression [11, 12], caregivers may find their time increasingly occupied with caregiving tasks to the negligence of other work and personal, pleasurable activities [13]. Such activity restriction has been shown to be related to caregiver depression [14]. Although the course of PD (including symptom development and intensity) and personal characteristics of caregivers and IWPDs may vary greatly, many informal PD caregivers experience mental health problems such as depression and anxiety at levels higher than the general population [7, 10].

However, one factor that has been found consistently in the research literature to buffer the development of depression from high levels of stress is one's perception of social support [15]. Social support has been shown to be negatively related to distress in general caregiving samples [16] as well as to feelings of burden in caregivers of IWPDs specifically [11, 17]. Although PD caregivers may perceive caring for their loved one as a duty they willingly undertake, many still report frustration at their own increasing isolation from friends and social activities as care burden intensifies [13]. Progression of the disease may necessitate the cessation of employment for both the IWPD and the family caregiver, reducing yet another avenue of social interaction and possible support [18]. Symptom manifestation can result in the IWPD feeling unable to cope with social situations and reluctance to interact with nonfamily members, so caregiver efforts to minimize stress for their loved one by agreeing with nondisclosure of the PD diagnosis or avoiding public situations may result in reduced social interactions for the caregiver as well as the IWPD [18].

It is therefore possible that social support in PD caregivers may be related to caregiver resilience. Resilience can be thought of as a response that is unusual for its adaptiveness in a difficult situation, allowing an individual to carry on with normal activities of life. For the purposes of the current study, Windle's [19] definition of resilience will be used.

“Resilience is the process of negotiating, managing, and adapting to significant sources of stress or trauma. Assets and resources within the individual, their life, and environment facilitate this capacity for adaptation and ‘bouncing back' in the face of adversity (p. 163).”

However, literature specifically examining resilience in PD caregivers is nonexistent. Contributing to the difficulty in studying resilience in PD caregivers is the heterogeneity of the disease course, the wide variety of personal characteristics of caregivers [20], the lack of a universally accepted definition of resilience as a construct [21], and the dearth of research that focuses exclusively on PD caregivers. It may also be the case that PD caregiver research has not yet made the transition from taking a risk factor-perspective to that of exploring personal strengths as protective factors. Much of what we know about caregiver resilience has been taken from studies conducted with caregivers of people with other neurodegenerative disorders like Alzheimer's disease. As PD may share symptoms with other neurodegenerative diseases (e.g., dementia), findings regarding caregiver resilience from such studies may also be relevant to caregivers of IWPDs. For example, a systematic review of resilience in caregivers of people with dementia found that depression is lower in caregivers possessing higher resilience and that social support, age, ethnicity, and sex are important factors in resilience levels [22].

Although many studies have examined various contributors to the mental health of caregivers generally, examination of this construct in PD caregivers represents a gap in the current literature. Therefore, this study employed a psychosocial perspective to explore the possible effects of two variables which have demonstrated relationships to mental health in numerous caregiver studies over the years, namely, social support (e.g., [16, 17]) and resilience (e.g., [22, 23]). Because only scant literature has focused on social support in PD caregivers and no studies to date have examined resilience in this population, the purpose of the current study was to construct and validate a theoretical structural equation model whereby social support is associated with resilience in PD caregivers, and increased resilience is related to decreased mental health symptoms. We hypothesized that all of the direct effects in this model would be statistically significant, as well as the indirect effect of social support on mental health through resilience.

## 2. Method

### 2.1. Participants

Informal caregivers for IWPDs were invited to participate in the current study while their care recipient was being seen at Parkinson's Clinic in the Hospital Civil Fray Antonio Alcalde, associated with the University of Guadalajara in Guadalajara, Mexico (*n* = 148) or at the Parkinson's and Movement Disorders Center of the Virginia Commonwealth University Medical Center in Richmond, Virginia, US (*n* = 105). These clinics were specifically chosen as data collection sites because both were situated in urban, public, academic medical centers within state capitals (i.e., Guadalajara, Jalisco or Richmond, Virginia). In order to be eligible for the study, participants needed to (a) be identified as the primary caregiver of a PD patient actively being seen in a medical clinic, (b) be at least 18 years of age, and (c) be fluent in either Spanish (for the Mexico site) or English (for the US site). The majority of the caregivers in this study were female (73.1%) spouses (68.8%) with a mean age of 59.92. Detailed participant demographic information and information on the individual living with PD collected from the caregiver by site and collectively are presented in Table 1.

### 2.2. Procedure

Following protocol review and approval by Institutional Review Boards at both sites, caregivers of individuals with PD were recruited through a variety of means including clinical visits, email, phone calls, flyers, direct contact, and word of mouth. Potential participants were given information regarding the study when they accompanied the patient to a medical appointment at one of the two clinics and provided informed consent. Caregivers were then screened for eligibility and, if eligible and consented, completed all study measures—a variety of paper-based surveys. Because of the clinic demographics and languages spoken by IWPDs and caregivers in the clinics, only a Spanish language option was provided for the Mexico site and an English option for the US site. Study measures were given orally at the Mexico site to allow for higher rates of illiteracy than in the US, while caregivers from the US completed all measures independently using pen and paper (since data collection methodology varied by site and thus could differentially have impacted measurement between the sites, Cronbach's alphas for each measure and site appear below.) Participants did not receive financial compensation for participating in the study. The data used in the current study were part of a larger data collection effort aimed at exploring demographic and psychosocial characteristics of PD caregivers in Mexico and the US. A more detailed comparison of Mexican and US PD caregiver and patient characteristics such as education, social class, and caregiving-related variables is outlined in another manuscript [24], which found that caregivers at the US site were older than those in Mexico, had higher education and social class, and spent fewer hours per week providing care.

### 2.3. Measures

#### 2.3.1. Social Support

The Interpersonal Support Evaluation List-12 (ISEL-12) was used to assess social support [25]. The ISEL-12 measures three components of social support: appraisal, belonging, and tangible. The Spanish version has shown good internal consistency across multiple Spanish-speaking samples [26]. Participants responded from “1” (definitely false) to “4” (definitely true) on questions like “When I need suggestions on how to deal with a personal problem, I know someone I can turn to.” All items were summed for a total score ranging 0–36, with higher scores indicating greater social support. Total score internal reliability for the current study was good (US *α* = 0.89, Mexico *α* = 0.80, and combined *α* = 0.84). Cronbach's alphas for the individual subscales were as follows: appraisal: US *α* = 0.83 and Mexico *α* = 0.65; belonging: US *α* = 0.85 and Mexico *α* = 0.59; tangible: US *α* = 0.71 and Mexico *α* = 0.48.

#### 2.3.2. Depression

The depression module of the Patient Health Questionnaire (PHQ), known as the PHQ-9, was used to assess depression [27]. The PHQ-9 has previously been validated in Spanish with good internal consistency [28–30]. Participants were asked to rate how often over the last two weeks they had been bothered by a problem such as “poor appetite or overeating” on a scale of “0” (not at all) to “3” (nearly every day). All items were summed for a total score ranging 0–27, with higher scores indicating greater severity of depression. Internal reliability for the current study was good (US *α* = 0.82, Mexico *α* = 0.81, and combined *α* = 0.82).

#### 2.3.3. Anxiety

Anxiety symptoms were assessed using the seven-item Generalized Anxiety Disorder-7 (GAD-7) scale [31]. The GAD-7 has previously been translated and validated in a Spanish version with good internal consistency [32]. Participants were asked to rate how often over the last two weeks they had been bothered by a problem such as “feeling nervous, anxious, or on edge” on a scale of “0” (not at all) to “3” (nearly every day). All items were summed for a total score ranging 0–21, with higher scores indicating greater severity of anxiety. Internal reliability for the current study was good (US *α* = 0.90, Mexico *α* = 0.88, and combined *α* = 0.88).

#### 2.3.4. Resilience

The Brief Resilience Scale (BRS) was used to assess resilience [33]. For the purpose of this study, the BRS was translated into Spanish and back-translated into English by bilingual and bicultural researchers. Discrepancies between the back-translated version and English version were addressed mutually by the translators. Participants were asked to rate on a scale from “1” (strongly disagree) to “5” (strongly agree), the degree to which they agreed with statements such as “I tend to bounce back quickly after hard times.” All items were summed for a total score ranging 6–30, with higher scores indicating greater resilience. Internal reliability for the current study was good (US *α* = 0.91, Mexico *α* = 0.88, and combined *α* = 0.89).

### 2.4. Model

**Figure 1:**
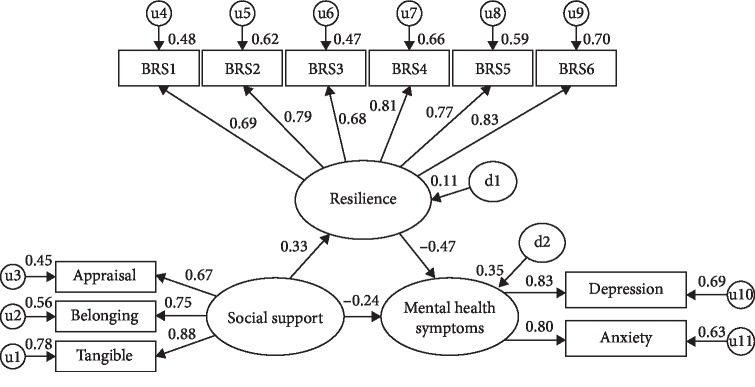
Structural equation model with standardized loadings. *Note.* BRS1-6 refers to items from the Brief Resilience Scale [33]; appraisal, belonging, and tangible are subscales of the Interpersonal Support Evaluation List-12 [25]; depression was measured by the PHQ-9 [27]; anxiety was measured by the GAD-7 [31].

A structural equation model (SEM) was developed using AMOS 26.0 [34]. The purpose of this SEM was to validate a hypothesized pattern of relationships among observed (manifest) and hidden (latent) variables leading from social support in informal caregivers of individuals with PD through resilience to mental health (depression and anxiety). The SEM model diagram with factor loadings (standard regression weights) appears in Figure 1. Latent variables are represented by the larger ovals (i.e., social support, resilience, and mental health), while the observed variables are represented by rectangles (e.g., depression, appraisal social support, and BRS1—a scale item of resilience). The most commonly used indices were chosen to assess the goodness of fit of the model [35, 36]. These indices and recommended cutoffs include a *χ*^2^ to degrees of freedom ratio of less than 2.0 [35]; 0.90 for the comparative fit index (CFI), the goodness of fit index (GFI), normed fit index (NFI), incremental fit index (IFI), Tucker–Lewis index (TLI), and adjusted goodness of fit index (AGFI) [37–39]; and the root mean squared error of approximation (RMSEA) of 0.08 or less [40]. Missing data in the current study were less than 1%, and maximum likelihood estimation was used.

## 3. Results

### 3.1. Correlation Matrix

All study measures of the primary variables under scrutiny were significantly correlated with one another (Table 2). The resilience total score was weakly positively correlated with the three subscales of social support (appraisal, belonging, and tangible) and moderately negatively associated with the total scores of depression and anxiety, suggesting that participants who endorsed higher degrees of resilience also scored higher on measures of social support and lower in measures of both depression and anxiety. The three social support subscales (appraisal, belonging, and tangible) were weakly negatively correlated with both measures of depression and anxiety, indicating that individuals with higher social support scores had lower scores for depression and anxiety. Depression and anxiety scores were both strongly positively correlated, demonstrating that people with higher measured depression were also likely to have higher measured anxiety.

### 3.2. Structural Equation Model

The overall fit for the model was generally good; *χ*^2^/d*f* = 2.05, CFI = 0.97, GFI = 0.95, NFI = 0.94, IFI = 0.97, TLI = 0.96, AGFI = 0.91, and RMSEA = 0.06. The model explained 11% of the variance in resilience and 35% of the variance in mental health symptoms. For the following statistics, *β* reflects a standardized beta and *b* reflects an unstandardized *b*-weight. In the model, social support was positively associated with resilience (*β* = 0.33, *b* = 0.16, SE = 0.04, and *p* < 0.001) and resilience was negatively associated with mental health symptoms (*β* = −0.47, *b* = −1.64, SE = −29, and *p* < 0.001). Social support also demonstrated a significant indirect effect on mental health through resilience (*β* = −0.16, *b* = −0.27, SE = 0.08, and *p* < 0.001); however, there was still a significant direct effect of social support to mental health symptoms (*β* = −0.24, *b* = −0.42, SE = 0.13, and *p*=0.008), suggesting a partial mediation. The total effect of social support on mental health symptoms was *β* = −0.40, *b* = −0.69, and *p* < 0.001, suggesting a notable drop in the size of the relationship between these variables with the addition of the mediator, resilience.

## 4. Discussion

The current study employed a strength-based perspective in order to construct and validate a theoretical structural equation model whereby social support is associated with higher levels of resilience in PD caregivers and increased resilience is related to decreased mental health symptoms. It was hypothesized that all of the direct effects in this model would be statistically significant, as well as the indirect effect of social support on mental health through resilience. This hypothesized pattern was robustly supported with the structural equation model showing generally good fit indices. Higher levels of social support were associated with increased resilience, which in turn was associated with reduced mental health symptoms. Resilience partially mediated social support's effect on mitigating mental health symptoms. The model explained 11% of the variance in resilience and 35% in mental health symptoms.

The positive relationship between social support and resilience has been well documented across the human lifespan, with evidence from psychological [41] as well as neurobiological research [42]. Studies with a wide variety of caregivers have also demonstrated the links between the social support rendered by family and friends and caregiver resilience [43]. For example, in maternal caregivers, social support has been negatively associated with distress [44] and in caregivers for individuals with Alzheimer's disease, social support was positively associated with resilience [23]. Studies looking at resilience in dementia caregivers have generally found that social support is an important factor in building resilience [22]. The current study's findings are the first to suggest that social support may be associated with increased resilience in PD caregivers.

The finding that resilience was negatively associated with mental health symptoms conforms to the patterns uncovered in previous research, such that resilience is known to be a protective factor for mental health generally [45], with higher levels of resilience associated with fewer symptoms of mental health problems and more indicators of good mental health [46]. In dementia caregivers, higher resilience has been associated with lower levels of depression [22]. This pattern was replicated in the current study, with PD caregiver resilience demonstrating a strong negative relationship to symptoms of depression and anxiety.

The most novel finding from the current study was that social support yielded a significant partial indirect effect on mental health through resilience in addition to its significant direct effect. Although research has shown that cancer caregivers who consistently feel a lack of good emotional support experience more depression over time [47], this is the first study finding that a possible mechanism in the relationship between social support in caregivers and mental health is via resilience, from a general caregiving perspective or with regard to PD specifically. Understanding this provides added targets for intervention, as both social support and resilience in PD caregivers may be bootstrapped clinically to assist with treatment of depression and anxiety.

The first variable initiating a cascade of salubrious effects on PD caregivers is social support, which is likely a critical intervention target. In order to improve social support, PD caregivers should be encouraged to take advantage of opportunities for respite from family members, friends, and professional providers. They should also be encouraged to form and maintain friendships and activities outside the care environment in order to increase resilience and foster supportive relationships [48]. Most informal PD caregivers are spouses or other family members [5, 6], so family relationship dynamics must also be considered when assessing the mental health impact of decreased social support for caregivers. Role changes and care responsibilities can add stress to interfamily relationships which may then lessen the support the caregiver has formerly received from family members (including the IWPD) [49–51]. The development and testing of family-based PD caregiving interventions that aim to improve family dynamics and thereby increase social support is an important area for future research.

The second variable in the structural equation model that could be an intervention target is resilience. A number of evidence-based resilience interventions have been developed for diverse patient and caregiver populations. For example, the Resilience and Adjustment Intervention [52] was developed for people with traumatic brain injury and targets adjustment challenges by emphasizing education, skill-building, and psychological support. A randomized clinical trial showed the intervention had a large-sized effect in increasing resilience. In a longitudinal study of Alzheimer caregivers, those who were randomly assigned to receive enhanced support through individual and family counseling and support-group participation showed significantly increased resilience throughout the caregiving time and even after the death of the care recipient [53]. Another caregiving intervention aimed to improve resilience in mothers of children with a recent diagnosis of cancer. Participants were randomly assigned to one of two conditions: problem-solving training or nondirective support. Both arms of the study improved resilience in the caregivers, but the problem-solving training arm showed evidence of continued growth postintervention [54]. Based on the findings of improvements in resilience among caregivers from these previous intervention studies, as well as the findings from the current study, it would be important that problem-solving, skill-building, and support interventions should be tailored specifically to PD caregivers to determine whether they improve caregiver resilience.

Finally, mental health symptoms such as depression and anxiety themselves can be directly targeted for intervention. Psychosocial interventions which have been successfully used to combat mental health symptoms in individuals with PD such as traditional and telephonically-administered cognitive behavioral therapy [55, 56] or group therapy [57] could be adapted for use in PD caregivers as well. In a metareview of interventions designed for dementia caregivers, social support, skill-building/educational, and problem-focused strategies for improving depression and other mental health outcomes were the interventions which most often showed significant effects [58]. As research in the area of improving PD caregiver mental health is still in its preliminary stages, future studies should utilize techniques which have been efficacious for caregivers of persons with other neurodegenerative conditions as a starting place.

Despite these implications for future PD caregiver intervention research, the current study had several limitations. First, the data are cross-sectional, and as a result, conclusive statements about causality in the mediational structural equation model cannot be made. Future studies employing cross-lagged panel or experimental designs should attempt to tease out causality more powerfully in the relationships among these variables. Second, data were collected via self-report from caregivers only, and no data were collected from individuals with PD or from healthcare providers. As a result, the data are susceptible to self-report biases, and observer reports of the constructs measured in the current study would be important in future research. Third, the measure of resilience used in the current study, though valid and well used in the literature, is a one-time snapshot of the construct. A stronger index of resilience would be to assess caregiver psychosocial functioning longitudinally and conceptualize it as a trajectory of positive adjustment over time. Fourth, the samples from Mexico and the United States had a fairly high degree of heterogeneity in terms of their demographic characteristics, as would be expected. Future research should more thoroughly examine potential differences between Mexico and the United States in terms of psychometric properties of the instruments used in the current study, of the relationships among the variables under scrutiny when controlling for potential demographic differences (which is challenging to do in an SEM) and of other demographics not considered (e.g., access to health services, caregiving resources, etc.). As a result, the model tested in the current study should be interpreted cross-culturally with an appropriate degree of caution.

## 5. Conclusion

Research in the area of PD caregiver psychosocial functioning is sparse, and the vast majority of caregiving studies have been conducted with caregivers for individuals with other neurological disorders like Alzheimer's disease. Findings from the current study have helped to elucidate the relationships between the extrinsic construct of social support and the intrinsic characteristic of resilience and their unique and collective influences on mental health in PD caregivers. Though PD presents unique challenges to those with the disease and their loved ones who are caring for them, results of the current study are congruent with the literature from other caregiving populations and suggest that social support is positively associated with resilience, which in turn is associated with mental health. Extrapolation of findings from intervention studies with other caregiving populations along with the findings from the current study may help in the development and tailoring of interventions to improve the resilience and mental health of PD caregivers.

## Figures and Tables

**Table 1:** Characteristics of PD caregivers and IWPDs.

Demographic variable	Mexico	US	Overall
CG age, years, mean *(SD)*	53.66 *(14.96)*	68.73 *(8.36)*	59.92 *(14.68)*
Patient age, years, mean *(SD)*	65.68 *(10.78)*	71.61 *(8.13)*	68.14 *(10.18)*
CG gender			
Man	35 (23.6%)	33 (31.4%)	26.9%
Woman	113 (76.4%)	72 (68.6%)	73.1%
Patient gender			
Man	77 (52%)	68 (64.8%)	57.3%
Woman	71 (48%)	37 (35.2%)	42.7%
Relationship to patient			
Spouse/significant other	76 (51.4%)	98 (93.3%)	68.8%
Parent	51 (34.5%)	4 (3.8%)	21.7%
Friend	1 (0.7%)	2 (1.9%)	1.2%
Sibling	11 (7.4%)	0	4.3%
Cousin	1 (0.7%)	0	0.4%
Aunt/uncle	2 (1.4%)	1 (1%)	1.2%
Other	6 (4.1%)	0	2.4%
CG race/ethnicity			
Latino/Hispanic	148 (100%)	0	58.5%
White/European (non-Latino)	0	97 (92.4%)	38.3%
Asian/Asian American/Pacific	0	3 (2.9%)	1.2%
Black/African American (non-Latino)	0	3 (2.9%)	1.2%
Multiracial/multiethnic	0	1 (1%)	0.4%
Other	0	1 (1%)	0.4%
CG highest completed education level			
Doctorate degree	0	8 (7.6%)	3.2%
Master's degree	3 (2%)	23 (21.9%)	10.3%
4-year college degree	24 (16.2%)	35 (33.3%)	23.3%
2-year/technical college degree	20 (13.5%)	12 (11.4%)	12.6%
High school/GED	8 (5.4%)	27 (25.7%)	13.8%
Elementary school	86 (58.1%)	0	34.0%
No formal schooling	7 (4.7%)	0	2.8%
CG employment status			
Full-time	18 (12.2%)	17 (16.2%)	13.8%
Part-time	42 (28.4%)	9 (8.6%)	20.2%
Unemployed	33 (22.3%)	6 (5.7%)	15.4%
Retired	9 (6.1%)	68 (64.8%)	30.4%
Student	1 (0.7%)	0	0.4%
Homemaker (Mexico only)	21 (14.2%)	0	8.3%
Other	24 (16.2%)	5 (4.8%)	11.5%
CG social class^*∗*^			
Upper	1 (0.7%)	3 (2.9%)	1.6%
Upper-middle	33 (22.3%)	67 (63.8%)	39.5%
Lower-middle	55 (37.2%)	25 (23.8%)	31.6%
Working	36 (24.3%)	10 (9.5%)	18.2%
Lower	23 (15.5%)	0	9.1%
Hours of care per week, mean *(SD)*	107.38 *(61.34)*	59.37 *(64.56)*	87.92 *(66.85)*
Months as a CG, mean *(SD)*	52.38 *(49.22)*	46.78 *(81.33)*	50.12 *(64.04)*
Months since PD diagnosis, mean *(SD)*	63.22 *(60.88)*	92.25 *(82.84)*	75.32 *(72.14)*

CG = caregiver. ^*∗*^For caregiver social class, participants were presented these exact options to select rather than a specific US dollar or Mexican peso amount in order to account for potential differences in buying power for the equivalent monetary amount in each country.

**Table 2:** Correlation matrix.

Variable	1	2	3	4	5
1 Resilience					
2 Appraisal SS	0.28^*∗∗*^				
3 Belonging SS	0.20^*∗∗*^	0.51^*∗∗*^			
4 Tangible SS	0.27^*∗∗*^	0.58^*∗∗*^	0.66^*∗∗*^		
5 Depression	−0.42^*∗∗*^	−0.25^*∗∗*^	−0.25^*∗∗*^	−0.33^*∗∗*^	
6 Anxiety	−0.44^*∗∗*^	−0.15^*∗*^	−0.13^*∗*^	−0.28^*∗∗*^	0.66^*∗∗*^

SS = social support. ^*∗*^*p* < 0.05; ^*∗∗*^*p* < 0.01.

## Data Availability

The data used to support the findings of this study are available from the corresponding author upon request.
